# Experimentally Verified Mathematical Model of Polymer Plasticization Process in Injection Molding

**DOI:** 10.3390/polym10090968

**Published:** 2018-09-01

**Authors:** Jacek Iwko, Ryszard Steller, Roman Wróblewski

**Affiliations:** 1 Department of Foundry, Plastics and Automation, Faculty of Mechanical Engineering, Wroclaw University of Science and Technology, Wybrzeże Wyspiańskiego 27, 50-370 Wrocław, Poland; r.m.wroblewski@pwr.edu.pl; 2 Department of Polymer Engineering and Technology, Faculty of Chemistry, Wroclaw University of Science and Technology, Wybrzeże Wyspiańskiego 27, 50-370 Wrocław, Poland; ryszard.steller@pwr.edu.pl

**Keywords:** injection moulding, plasticizing system, mathematical model, verification

## Abstract

The mathematical model of the polymer plasticization in the reciprocating screw injection moulding machine is presented in this paper. Methods of calculation of the most important flow characteristics, such as the solid bed profile, the pressure and temperature profiles, the mass flow rate, the power demand, the screw torque and the energy consumption were analysed. According to the mathematical model, a computer program was developed. Based on the computer program, simulation studies of the injection moulding process were conducted. Thereafter, the experimental studies, evaluating the theoretical model from the accuracy and usefulness point of view, were carried out. Important output quantities, such as the temperature and pressure profiles, the power demand by the screw, the torque on the screw and the screw rotation time were measured. The studies were performed on a specially made research office. The simulation results were compared with the experimental data measured for the most popular polymers and different operating parameters of the injection machine. The experimental studies have indicated the need to introduce some corrections to the mathematical model. Several modifications have been made to the model, mainly related to the methods of stress determining in the polymer layer. Finally, the output characteristics of the plasticization process in the injection moulding are now correctly determined by the model with an average error less than 10%.

## 1. Introduction

One of the main factors that minimizes the production costs is the optimal choice of the processing equipment and processing conditions. For a long time, the optimization of geometry of plasticizing systems and forming tools in the injection moulding process have used the experience of designers and manufacturers. Recently, the theoretical approach has been having the increasing importance. It relies on using mathematical models for the plasticization process on the basis of the law of mass, momentum and energy conservation and the characteristics of a material. The models join the output characteristics of the plasticization process, such as pressure and temperature distribution, throughput, power demand and so forth, with the geometry of the plasticizing system, the adjustable process parameters and the material data, allowing thereby the optimization of the equipment design [1].

The theoretical approach to the plasticization process through the creation of computer-based simulation models is widely used mainly in the case of the extrusion process. Many models can be found in the literature. They describe, in less or more complex way, the plasticization of polymers in single-screw extruders. These models commonly use similar principles but differ in detailed assumptions. Thus, the extruder is divided into three main functional zones—the zone of solid conveying, the transient zone and the zone of melting and melt conveying. The description of the solid conveying zone is based on the dry friction mechanism represented usually by the classic approach of Darnell and Moll [2] with subsequent modifications [3,4,5,6,7]. It could also include grooves on the barrel in isothermal and non-isothermal conditions. The models describing the solid polymer conveying based on the description of granular systems have also appeared recently. They use 3-D discrete particle simulations [8]. The existing models were also subsequently reviewed in various monographs related to the polymer extrusion [3,9,10,11].

The existing models of the polymer melting in the screw systems can be divided into two categories, that is, the models of contiguous solid melting (CSM) and the models of dispersed solid melting (DSM). The Tadmor (CSM) model [3] and its various modifications are used most commonly [12,13,14,15,16,17] for the description of the melting process in single-screw systems. This model assumes that the melting occurs in a thin layer between the heated barrel and the solid polymer bed which moves with a constant velocity along the screw channel. The circulated melt pool, appearing at some critical thickness of the melting layer, accumulates at the active screw channel flight. Its relative width increases gradually dependent on the process parameters and the screw geometry. Modifications of the Tadmor model take into account such phenomena as changes of the solid bed velocity, the solid bed breakup or melting accompanied by the circulating melt flow around the solid bed. Flat or cylindrical screw channel shape and one- or two-dimensional, non-isothermal flow are usually assumed. The rheological properties of the polymer melt are described as a rule by the power law, Cross and Carreau models. It allows the calculation of typical characteristics of the process, depending on the channel geometry and the operating conditions. The models using a multi-dimensional finite element simulation are also available [18].

The DSM melting mechanism is characteristic for the starve-fed extrusion regime, which is more relevant of the twin-screw extrusion. The starve-fed conditions in single-screw extruders can be obtained with a controlled feeding, because for typical processes the flood feeding described by the CSM models prevails. Although the starve-fed in the single-screw extruders has been known for a long time [19,20], its systematic study and modelling have begun in the last decade [21,22,23].

In the last two decades, there has also been observed a significant development in the field of modelling of the plasticization process in the twin-screw extruders, both in the co-rotating systems [24,25,26,27] as well as in the counter-rotating systems [28,29,30].

Despite the significant development of the extrusion simulation programs, only very few simulation models describe the plasticization process in the injection moulding. The main cause is a much more complex dynamic of the plasticization process resulting from the cyclical nature of the injection moulding. It involves the existence of coupled static and dynamic melting phases (stationary and rotating screw) accompanied by an axial screw movement with an adjustable stroke. The model approach to melting mechanism in the injection moulding has been the subject of a few works [17,31,32,33,34], without further continuation of the model development. However, in the last two decades several new works have been published [35,36,37,38,39,40,41,42,43,44,45]. A paper outlining the calculation of the power requirement of the plasticizing systems of the injection moulding machines and the extruders was introduced by Potente [39]. He also presented the mathematical approach to simulate the polymer plasticization process in the injection moulding [40]. However, this model does not take into account the solid conveying and transient zones and uses some special modelling empirical constants. There are also reports on the experimental study of the solid bed width in the screw channel of the injection moulding machines [41,42] with the use of “transparent windows” made in the barrel to observe the behaviour of the solid polymer and the plasticization process. A few years ago, a new model for the plasticization process of the injection moulding was presented [43]. This model takes into account the backward movement of the screw, the presence of a non-return valve and the conduction of the heat during the idle time. Another comprehensive model of the plasticization process in the injection moulding which reflects well the dynamics of a real reciprocating screw was presented in [44,45]. More details of this model were published in [1,46,47].

It follows from the foregoing literature data that the creation of an adequate and comprehensive simulation model of the plasticization process in the injection moulding has not been fully completed yet. It confirms that there exist at least several commercial software programs, such as EXTRUD, SSD, REX, SSEM and NEXTRUCAD for the extrusion process but there is probably one computer program—PSI available for the analysis of the plasticization process in the injection moulding.

The purpose of this article is to present the comprehensive model of the polymer plasticization process in the injection moulding. The previous version of this model and the simplified model verification (limited only to comparison of the screw rotation time) was presented in [44,45]. Next, the experimental research which evaluates the theoretical model from the accuracy and usefulness point of view, was conducted. Important output quantities such as the temperature and pressure profiles of the polymer, the power demand of the plasticizing system, the torque of the screw and the recovery time were measured. These tests were performed on a specially designed research office. The experimental studies indicated the need for introduction of some corrections to the mathematical model. Consequently, several modifications were made in the model. The changes were related to the methods of stress determining in the polymer layer in the screw-barrel system. Furthermore, another method for determining the temperature of the molten polymer in a slit between the top of the screw flight and the barrel was indicated. The above two groups of changes resulted in a significant improvement in determining the power demanded to the screw and the torque on the screw in the transition and melting zones. This is the most important change in this model compared to the model described in [44]. The method for determining the time of the full injection moulding cycle was also improved. Thanks to these modifications, the output characteristics of the plasticization process in the injection moulding are now correctly determined, with an average error less than 10%. The full mathematical model and a brief summary of the results of its experimental verification is presented below.

## 2. Mathematical Model

The model description is comprised of three parts. The first one consists of definitions and main assumptions. Then, the key segments of its construction are presented. The last part of the description discusses the calculation procedure.

**Figure 1:**
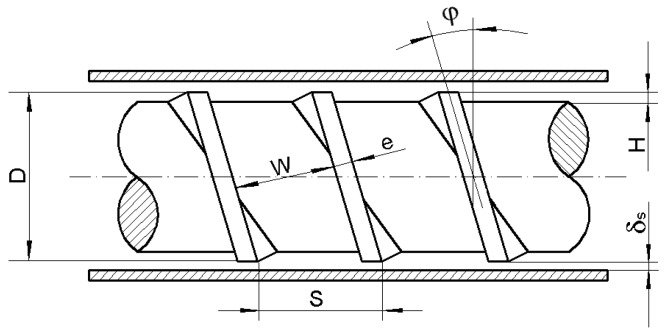
Geometry of the screw-barrel system of the injection screw machine.

The plasticizing unit shown in Figure 1 consists of a barrel with a feed hopper, a heating section and a three-zones-screw of diameter *D*, width *W* (constant on the whole screw length), lead *S* and flight width *e*. The channel depth *H* in feed and metering zones are constant and equal to *H_f_* and *H_m_*, respectively. The channel depth in a compression (transition) zone changes linearly from *H_f_* to *H_m_*. The additional geometric parameters of the model are flight clearance *δ_S_* (thickness of the slit between the top of screw flight and the inner barrel surface) and the lengths of feed, compression and metering zones. The helix angle *ϕ* depicted in Figure 1 can be calculated for known values of *D* and *S*. It is also possible to analyse flows in two or more parallel aligned channels. The above geometric parameters are principally the same as those used in various extrusion models.

In order to create a mathematical model of the plasticization process during the injection moulding, several general assumptions were made.

They are the following:

(a) The injection moulding is a quasi-steady process, that is, the process characteristics such as the pressure, temperature, solid polymer and melt profiles change with time during a single injection cycle but they are constant for the same moments of time in different cycles.
(b) The plasticization during the injection moulding in contrast to the extrusion contains two different and strictly coupled melting phases–static melting at stationary screw and dynamic melting at the rotating screw and its axial backward movement.
(c) For the assumed screw geometry and adjustable operating parameters (e.g., the rotational velocity, screw stoke, barrel temperature, dwell time, back pressure, etc.) the screw retreat is generally determined by the equality of the calculated pressure in the front of the screw and the pre-set back pressure.
(d) The most important process characteristics correspond to the moment of time just before the end of the screw rotation, that is, to the maximal filling degree of the screw channel with the mixture of solid polymer and melt.
(e) Description of various phenomena connected with the plasticization process, for example, the solid polymer and melt transportation, the static/dynamic melting mechanism, non-isothermal flow conditions and so forth, requires additional detailed assumptions discussed below.

**Figure 2:**
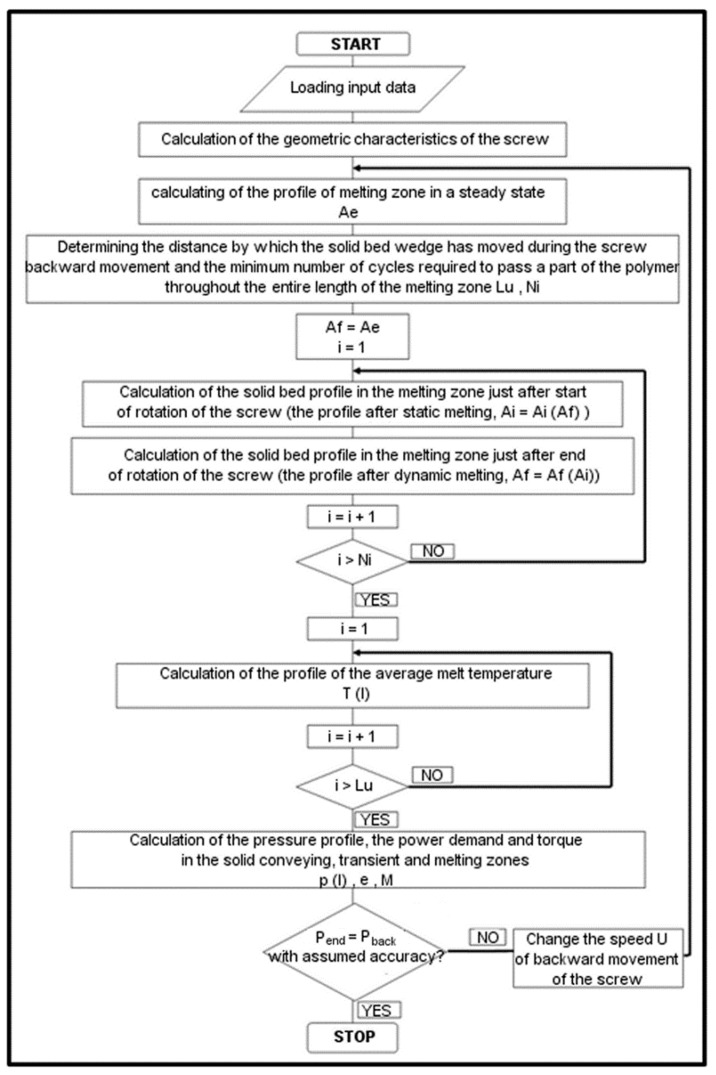
The algorithm of the computer program, based on the presented model.

Figure 2 presents the general calculation algorithm of the computer model based on the above assumptions.

It can be seen that the algorithm comprises three loops. The largest loop is connected with the pressure calculations according to the assumption (c). This makes it possible to calculate the screw backward movement velocity *U* (used for calculations of the other quantities). The first smaller loop calculates the coupled (time dependent) solid bed profiles after static melting and dynamic melting according to the assumption (b). It uses the assumption that the profile after the static melting is the initial profile for the dynamic melting and inversely. The third loop calculates the time dependent melt temperature changes during the dynamic melting assuming that the initial melt temperature after the static melting is approximately constant and equal to the barrel temperature. Other calculation details are discussed below.

Firstly, the existence of three dynamical zones in the plasticizing system was assumed:

1. a feed port and a solid conveying zone;
2. a transient (delay) zone;
3. a melting and melt conveying zone.

Furthermore, the flat (rectangular) screw channel model was assumed.

The starting point for the model is the model of steady-state extrusion that is similar to the classical extrusion model of Tadmor and Klein [3]. However, in contrast to the steady conditions characteristic for extrusion, the lengths and positions of dynamical zones change in time within the injection cycle. To describe these time changes it was adopted, that two coupled states (appearing at two characteristic moments of time) are valid during the cycle:

- at the end of screw rotation (the beginning of static melting);
- at the beginning of screw rotation (the beginning of dynamic melting).

Moreover, it was assumed that the melt behaviour can be described by the power-law of the form:

(1) $$\tau =k_{0}e^{-a\left(T-T_{o}\right)}{\left(\frac{1}{2}II_{d}\right)}^{n-1}d$$

where *τ*—the extra stress tensor, *d*—the rate-of-strain tensor, *II_d_*—the second invariant of *d*-tensor, *T_0_*—the reference temperature (usually assumed as the polymer melting temperature), *k*_0_, *n*, *a*—rheological parameters: *k*_0_—the consistency coefficient, *n*—the power-law exponent, *a*—the temperature coefficient.

### 2.1. Solid Conveying Zone

**Figure 3:**
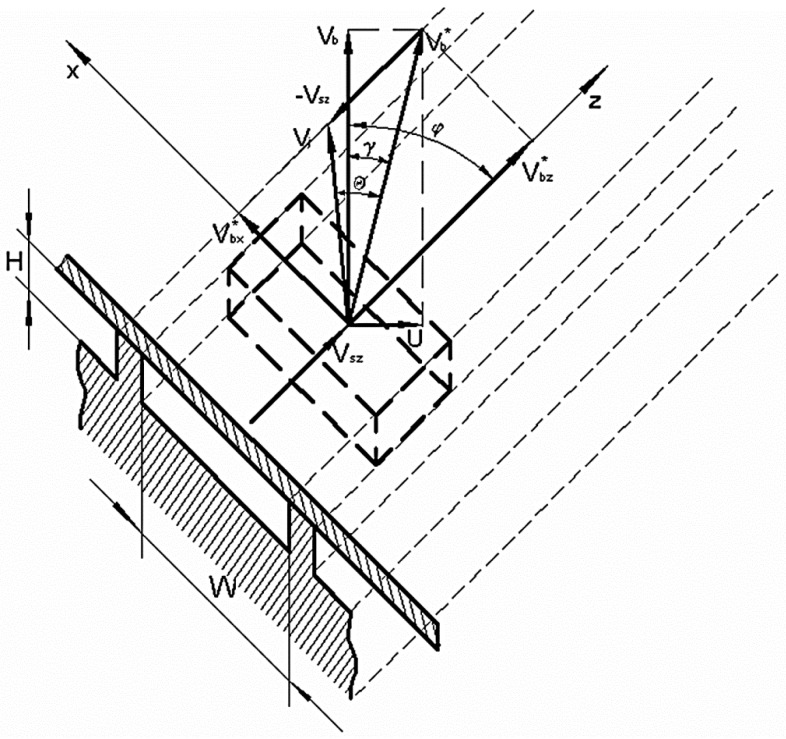
The mechanism of solid conveying in the injection screw channel.

It was assumed that the dynamic equilibrium in the solid conveying zone is established fast enough. Hence, its operating characteristics can be adequately described by means of relations, that are valid for the steady-state conditions [3]. However, the axial velocity component *U* of rotating and withdrawing screw should be additionally taken into account. The generalized mechanism of solid conveying with axial screw motion is shown in Figure 3.

Assuming the flow continuity, the mass flow can be calculated both from the solid bed velocity and from the screw withdraw velocity as

(2) $$\dot{G}=HWV_{sz}\rho_{s}$$

(3) $$\dot{G}=\frac{1}{4}\pi D^{2}U\rho_{m}$$

where *H*—the channel height, *W*—the average channel width, *V_sz_*—the solid bed velocity along the screw channel (in *z*-direction), *ρ_s_*—the density of solid polymer, *D*—the outer screw diameter, *U*—the axial screw velocity, *ρ_m_*—the average density of polymer melt; The *V_sz_* velocity is determined from Figure 2 [44].

If the mass flow $\dot{G}$ is known, the values of *V_sz_* and *U* can be calculated and this makes possible to calculate the solid conveying angle *θ* from (4). If this angle is known, the pressure profile in the solid conveying zone can be determined using the force and torque balance [2,44]. A general equation describing the pressure changes over the zone length has the form:

(4) $$p_{2}=p_{1}exp\left(k\Delta \right)$$

where *k*—the parameter determined from the balance of forces and moments of force acting on the material layer of elementary thickness *dz* [2], ∆—the length of one computational step in *z*-direction.

The initial pressure *p*_0_ in the feed port region can be calculated according to the simple formula proposed in [34]:

(5) $$p_{0}=\rho_{0}gD$$

where *ρ*_0_—the bulk density, *g*—the gravitational acceleration.

For the given pressure profile, it is possible to determine the power demand *e_S_* in the solid conveying zone as the sum of power dissipated at the barrel, screw root, screw flights and the power used to increase the pressure in the solid bed:

(6) $$e_{s}=p\left(l\right)f_{b}\;W\;\Delta \;V_{j}+p\left(l\right)f_{s}\;W\;\Delta \;V_{sz}+2p\left(l\right)\;f_{s}\;H\;\Delta \;V_{sz}+H\;W\;V_{sz}\frac{dp}{dl}$$

where p(*l*) is the pressure in *l*-location; *l* is the location on the screw channel length (in *z*-direction) measured by a number of computation steps from the beginning of the screw; *f_b_*, *f_s_*—the barrel and screw friction factors of solid polymer, respectively, *V_j_*—the velocity of solid bed transport relative to the barrel, determined from Figure 3 [44].

The torque *M_S_* in the solid conveying zone was determined in the classical way as the product of the dry friction force and the lever arm:

(7) $$M_{s}=p\left(l\right)\;f_{b}\;W\;\Delta \;\frac{D}{2}$$

From the presented relations, it follows that the main difference in description of solid conveying zone action during extrusion and injection moulding is the existence of the non-zero retraction velocity of the screw.

### 2.2. Transient Zone

The transient zone in the model starts at a point, where melt layer appears at the solid bed surface. It was adopted, that this is the place of the screw channel which corresponds with the beginning of the barrel heating zone at a given moment of time. The end of the transient zone in the screw channel corresponds with the point, where the melt film thickness reaches a critical value *δ_w_* [3]. In contrast to the solid conveying zone, the total length of the transient zone is variable and it depends on the process conditions. The length of this zone is very short and it usually reaches half to two coils. However, it is important to consider this zone, because it allows continuity of the pressure profile as well as the correct calculation of the total power demand and torque on the screw. According to [3] it was assumed that the melt film thickness changes linearly from 0 to *δ_w_* over the zone length. These changes depend on the rate of dynamic melting, that can be calculated from [3], assuming additionally the axial screw velocity *U* [44].

The calculations of the pressure changes in the transient zone base on the assumption that the pressure gradient in this zone can be determined as a weighted average of the pressure gradient at the end of the solid conveying zone and the pressure gradient at the beginning of the melting zone:

(8) $${\left(\frac{\partial p}{\partial z}\right)}_{t}={\left(\frac{\partial p}{\partial z}\right)}_{s}\;\left(1-x\right)+{\left(\frac{\partial p}{\partial z}\right)}_{m}\;x$$

where the subscripts *t*, *s* and *m* mean: the transient zone, the solid conveying zone and the melting zone, respectively, *x*—the weight factor, changing from 0 to 1 on the length of the transient zone.

This semi empirical approach that provides a smooth pressure profile at the zone boundaries was introduced, because there is no exact method of pressure calculation in the case, if the flow is determined by both dry and viscous friction.

The power demand *e_T_* in the transient zone was defined as the sum of the power dissipated in the thin melt film at the barrel surface, on the root surface and the flight surfaces, as well as the power desired to increase the pressure. It can be calculated for one computation step from the equation:

(9) $$e_{T}=\left[p\left(l\right)f_{b}\left(1-x\right)+\tau_{j}\;x\right]\;W\;\Delta \;V_{j}+p\left(l\right)f_{s}\;W\;\Delta \;V_{sz}+2\;p\left(l\right)f_{s}\;\left(H-\delta_{m}\right)\;\Delta \;V_{sz}+H\;W\;V_{sz}\frac{dp}{dl}+\tau_{e}\;e\;\Delta \;\sqrt{V_{b}^{2}+U^{2}}$$

where *τ_j_*—the shear stress in the polymer melt layer in the screw channel, *δ_m_*—the melt layer thickness in the screw channel in the transient zone, changing linearly from 0 to critical value *δ_w_*: *δ_m_* = *δ_w_ x* (*x*—the weight factor), *τ_e_*—the shear stress in the polymer melt layer in the slit between the top of the screw flight and the barrel surface, *e*—the width of the screw flight; *τ_j_* and *τ_e_* quantities are defined according to the power law as follows:

(10) $$\tau_{j}=k_{0}\exp\left(-a\;\left(T_{b}-T_{m}\right)\right){\left(\frac{V_{j}}{\delta_{m}}\right)}^{n}$$

(11) $$\tau_{e}=k_{0}\exp\left(-a\;\left({\bar{T}}_{slit}-T_{m}\right)\right){\left(\frac{\sqrt{\left(V_{b}^{2}+U^{2}\right)}}{\delta_{s}}\right)}^{n}$$

where *k*_0_, *a*, *n*—the parameters of the power law equation, *δ_s_*—the slit thickness, *T_b_*—the average barrel temperature in the heated zone, *T_m_*—the melting (flow) temperature of polymer, ${\bar{T}}_{slit}$—the average polymer temperature in the slit.

The first term in Equation (9) assumes the occurrence of the polymer-barrel friction as a weighted average of dry (*p* (1 − *x*)) and viscous (*τ·x*) friction. The last term of the Equation (9) refers to the power dissipated in the thin polymer layer existing between the top of the screw flight and the inner barrel surface as a result of the leakage flow. The determination of the polymer temperature in the slit bases on an energy equation, which assumes neglecting of the convection and the conductivity along and across the slit for the generalized Newtonian liquid and takes the form:

(12) $$\rho_{m}c_{m}\frac{\partial T}{\partial t}=k_{m}\frac{\partial^{2}T}{\partial y^{2}}+\eta {\left|\vec{\dot{\gamma }}\right|}^{2}$$

Averaging on the slit thickness, the Equation (12) takes the form:

(13) $$\frac{\rho_{m}c_{m}}{\delta }\frac{\partial \int_{0}^{\delta }Tdy}{\partial t}=\frac{k_{m}}{\delta }\left[{\left(\frac{\delta T}{\delta y}\right)}_{y=\delta }-{\left(\frac{\delta T}{\delta y}\right)}_{y=0}\right]+\frac{\int_{0}^{\delta }\eta dy}{\delta }{\left|\vec{\dot{\gamma }}\right|}^{2}$$

Due to the high intensity and the short shear time as well as the partial compensation of thermal effects at the surface of the barrel and the flight it is assumed that the flow is approximately adiabatic. Thus, the Equation (12) assumes the simplified form:

(14) $$\rho_{m}c_{m}\frac{\partial \bar{T}}{\partial t}=\bar{\eta }{\left|\vec{\dot{\gamma }}\right|}^{2}$$

wherein, according to the power law equation, the average viscosity can be defined by the formula

(15) $$\bar{\eta }=k_{0}\cdot e^{-a\left(\bar{T}-T_{m}\right)}{\left|\vec{\dot{\gamma }}\right|}^{n-1}$$

**Figure 4:**
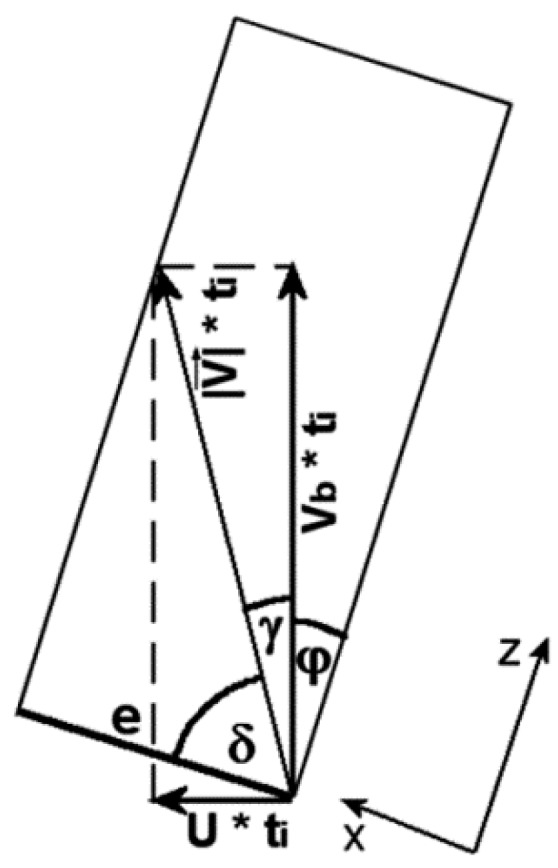
The vector analysis of the polymer particle displacement in the slit.

Analysing the displacement of the polymer particle in the slit (see Figure 4), the shear time *t_i_* in the slit is equal to the transition time from one to the other edge of the slit with the width *e* and the helix angle *ϕ* in the direction and the velocity determined by the vector $\vec{V}$. The shear time can be easily calculated on the basis of the geometrical relationships from Figure 3 as:

(16) $$t_{i}=\frac{e}{\left|\vec{V}\right|\sin\left(\varphi +\gamma \right)}$$

where $\left|\vec{V}\right|=\sqrt{V_{b}^{2}+U^{2}}$.

The Equation (14) with the viscosity expressed by the Formula (15), integrated with the initial condition $\bar{T}\left(t=0\right)=T_{b}$ and taking into account the Formula (16) allows to obtain the expression which describes the polymer temperature in the slit:

(17) $${\bar{T}}_{slit}=T_{b}+\frac{1}{a}ln\left[\frac{k_{0}\;a\;e^{-a\left(T_{b}-T_{m}\right)}}{\rho_{m}\;c_{m}\;sin\left(\varphi +\gamma \right)}{\left|\frac{\vec{V}}{\delta_{s}}\right|}^{n}\frac{e}{\delta_{s}}+1\right]$$

where *c_m_*—the average specific heat of the polymer melt.

The torque *M_T_* in the transient zone was determined as:

(18) $$M_{T}=\left[p\left(l\right)\;f_{b}\;\left(1-x\right)+\tau \;x\right]\;W\;\Delta \;\frac{D}{2}$$

where *τ* is the shear stress defined as:

(19) $$\tau =k_{0}\exp\left(-a\;\left(T_{b}-T_{m}\right)\right){\left(\frac{V_{b}}{\delta_{m}}\right)}^{n}$$

In conclusion, it should be noted that the transient zone could not be omitted from the model calculations, as it is in the case of some other available models. This is due to the experimental studies that indicated this zone is necessary for obtaining the correct values of the output characteristics. It is also important that the weighted average of dry friction and the viscous friction at the polymer-barrel contact should be maintained in the transient zone. Adopting viscous friction only results in significant overestimation of the pressure, power and torque characteristics.

### 2.3. Melting Zone

The melting process during the injection moulding is more complicated in comparison with the extrusion, mainly due to the existence of the static melting phase (for the stationary screw). Moreover, the phase of the dynamic melting must additionally take into account the axial screw motion. Both phases are coupled. The final conditions for one of them are the initial conditions for the second one.

Defining of the function of the solid bed width distribution in time and space (along the screw channel) is the basis for the plasticization model with the three-zone-screw. It can be described with the general relation:

(20) $$\frac{X}{W}=f\left(l,t\right)$$

The spatial coordinate *l* (along the screw channel) in the Equation (20) is expressed in the number of computation steps.

It was assumed that three areas of different behaviour of the polymer material, shown in Figure 4, can be distinguished in the screw channel cross section in the melting zone [3,32]. There is an area occupied by a homogeneous block of solid polymer, a melted polymer layer at the barrel surface and a melt pool.

**Figure 5:**
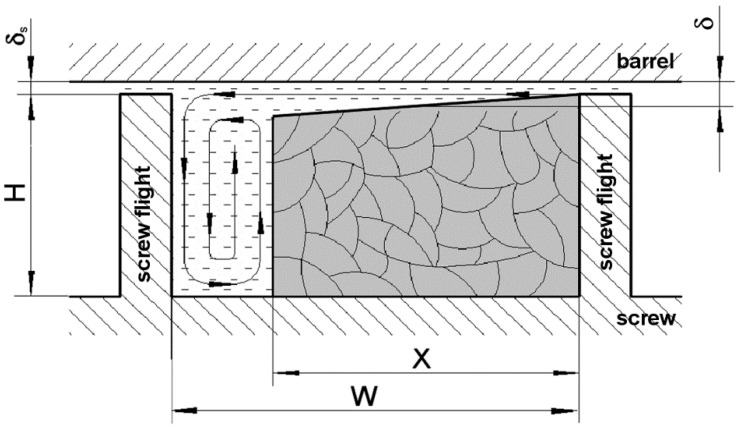
The screw channel cross-section in the melting zone (*δ*—average thickness of the polymer melt layer).

Due to the variable channel height along the screw it is convenient to express the solid bed profile as the function

(21) $$A\left(l,t\right)=H\left(l\right)\frac{X\left(l,t\right)}{W}$$

where *A*(*l*,*t*)—the ratio of the cross-sectional area of the channel occupied by the solid bed to the total cross-sectional area of the channel (see Figure 5), *H*(*l*)—the relative height of screw channel, determined as *H*(*l*) = *H*/*H_f_*, changing from 1 (in the geometrical feed zone) to *H_m_*/*H_f_* (geometrical metering zone) on the screw channel length.

Knowing the *A*(*l*) function in the two basic moments of time, is necessary to describe of the melting process in the injection moulding:

1. just after the start of the screw rotation (beginning of dynamic melting): $A\left(l,0\right)=A_{i}\left(l\right)$,
2. just after the finish of the screw rotation (beginning of static melting): $A\left(l,t_{r}\right)=A_{f}\left(l\right)$, where *t_r_* is the recovery time defined as:
  (22) $$t_{r}=\frac{N_{s}\;S}{U}$$
  where *N_S_* is the screw stroke (expressed in number of coils).

The static melting begins after the stopping the rotational screw motion. The solid polymer is molten in a certain time interval, which is approximately equal to the cooling time and then the screw is shifted forward on the distance of the screw stroke. The polymer is molten now in the time approximately equal to the hold time. The method of calculating the solid bed profile after the static melting process is given in [44].

Dynamic melting starts at the moment of the beginning of the screw rotation. The calculation of the solid bed profile after the screw rotation period was done using the theory of dynamic extrusion [31]. The method of calculating the solid bed profile after the dynamic melting process is given in [44].

The quantity *L_u_* defined by Equation (23) describes the solid bed displacement due to the rotary-backward motion of the screw and it is expressed in the computation steps. The solid bed evolution during the screw rotation can be followed by changing *L_u_* from 0 to the end value given by the Equation (23).

(23) $$L_{u}=\frac{t_{r}\;V_{sz}}{\Delta }=\frac{N_{5}\;S\;V_{sz}}{U\;\Delta }$$

Equilibrium values of A after the static and dynamic melting can be calculated using the iteration method. The steady-state profile *A_e_* characteristic for the extrusion process [3] taking additionally into account the backward screw motion could be assumed as the first approximation of A [44] (theoretically any profile could be taken into account). Hence, the approximated *A_i_* profile after static melting can be determined. It is the initial value for the new profile of the solid bed *A_f_* calculated for dynamic melting. The iteration is repeated until *A* profiles (after static and dynamic melting) will be established. It could be shown that the time required for stabilization of *A_f_* and *A_i_* is not shorter than the passage time of the first polymer portion over the whole channel length. It corresponds to a certain value of *N_i_* iteration cycles [44].

If the solid bed profiles are known, the pressure and temperature profiles in screw channel can be calculated. Knowing these profiles makes it possible to calculate other quantities: the power demand, the screw torque and the energy consumption that are important for the detailed characterization of the plasticization process. All the quantities were calculated for the *A_f_* profile after the dynamic melting, which is characterized by the maximal filling of the screw channel with the solid polymer.

The temperature profile is the result of thermal processes during the whole screw rotation phase. The methods of polymer melt temperature calculation, which are valid for the steady-state conditions, could not be applied for the calculation of the temperature profile in the injection moulding. In this case, the temperature profile was determined by an approximated method described in [34], which was adapted to the model requirements. The method of calculating the temperature profile of the molten polymer in the screw channel, used in the model, was presented in [44].

For the pressure calculation, we have assumed that the polymer pressure in the screw channel is stabilized fast enough. Hence, for its calculations the same methods can be used as for the steady-state conditions. The pressure was calculated according to the own method based on the results of the analysis of the two-directional, non-isothermal flow of the Ellis fluid in the rectangular channel [48]. The exact method of calculating the pressure profile in the screw channel, used in the model, was presented in [44].

The total power demand in melting zone *e_M_* is the sum of the power dissipated in the melt region due to the longitudinal and transversal flow, the power dissipated in the slit between top of screw flight and the barrel inner surface and the power required for pressure changes:

(24) $$e_{M}=\tau_{z}\;W\;\Delta \;V_{bz}^{*}F_{d}+\tau_{x}\;W\;\Delta \;V_{bx}^{*}+\tau_{e}\;e\;\Delta \;\sqrt{V_{b}^{2}+U^{2}}+H\;W\;\bar{V_{mz}}\;\frac{dp}{dl}$$

where

(25) $$\tau_{z}=k_{0}\exp\left[-a\left(\bar{T}-T_{m}\right)\right]\;\left(\frac{V_{bz}^{*}\;F_{d}}{H}\right)$$

(26) $$\tau_{x}=k_{0}\exp\left[-a\left(\bar{T}-T_{m}\right)\right]\;\left(\frac{V_{bx}^{*}}{H}\right)$$

*τ_e_*—the shear stress in the polymer melt layer in the slit, defined by Equation (11).

The torque *M_M_* in the melting zone was determined as a sum:

(27) $$M_{M}=\tau_{xz}\;W\;\Delta \;\frac{D}{2}+\tau_{e}\;e\;\Delta \;\frac{D}{2}$$

where *τ_xz_* and *τ_e_* are defined respectively as

(28) $$\tau_{xz}=\sqrt{\tau_{x}{}^{2}+\tau_{z}{}^{2}}$$

(29) $$\tau_{e}=k_{0}\exp\left(-a\;\left({\bar{T}}_{slit}-T_{m}\right)\right){\left(\frac{V_{b}}{\delta_{s}}\right)}^{n}$$

#### The Calculation Procedure

One of the most fundamental questions in the model is the determination of the screw retraction velocity *U* and the pressure profile, where the pressure value at the screw end is equal to the known back pressure (operating parameter). Both quantities are strictly coupled and their determination closes the computation cycle. Hereafter, that makes it possible to calculate the most important process characteristics such as the plasticization rate, the power requirement, the screw torque, the average melt temperature and the specific energy consumption. The choice of the proper backward velocity U for a given back pressure was done with the iteration method using a special control algorithm. It increases or decreases the *U* value depending on the calculated pressure on the screw end and the assumed back pressure, until both pressures become equal with a desired accuracy. The algorithm adopts the following form:

(30) $$U_{2}=U_{1}{\left(1+\frac{p_{end}-p_{back}}{\left|p_{end}-p_{back}\right|+p_{back}}\right)}^{sgn\left(p_{end}-p_{back}\right)}$$

In summary, some earlier concepts of the modelling of the plasticization process in the injection moulding have been taken into account. Due to the partial similarity between the extrusion and the reciprocating injection moulding, some solutions from the existing extrusion models have also been applied. However, many other issues are the original solutions: the generalization of the static and dynamic melting process for the three-zone screw, the description of reciprocating screw motion with the adjustable stroke, the description of two-dimensional, non-isothermal and non-Newtonian (power-law) melt flow in the channel, the solution for the time dependent mass balance during the dynamic melting and the description of the instant melt temperature changes [44,48,49].

The full operation algorithm of a computer program based on the model shown above is presented in Figure 2. The model uses four groups of input data: the geometric parameters of the three-zone screw and the barrel, the adjustable operating parameters of the injection moulding machine, the material data and the numerical data (rate and accuracy of calculations). The following characteristics of the plasticization process can be determined: the relative solid bed width, the pressure and temperature profiles of the polymer along the screw, the torque and the recovery time, the power demand, the throughput and the SEC (specific energy consumption).

For evaluation of prediction accuracy and possible improvements, the simulation model required a full experimental verification based on the comparison of its output characteristics mentioned above with the experimental characteristics of the real plasticizing unit. For this purpose, the measuring system as the special equipment connected with the conventional injection moulding machine was designed and built [1,50]. Selected results of the comparison of the results generated by the model with the experimentally determined process characteristics are presented below.

## 3. Research Office

**Figure 6:**
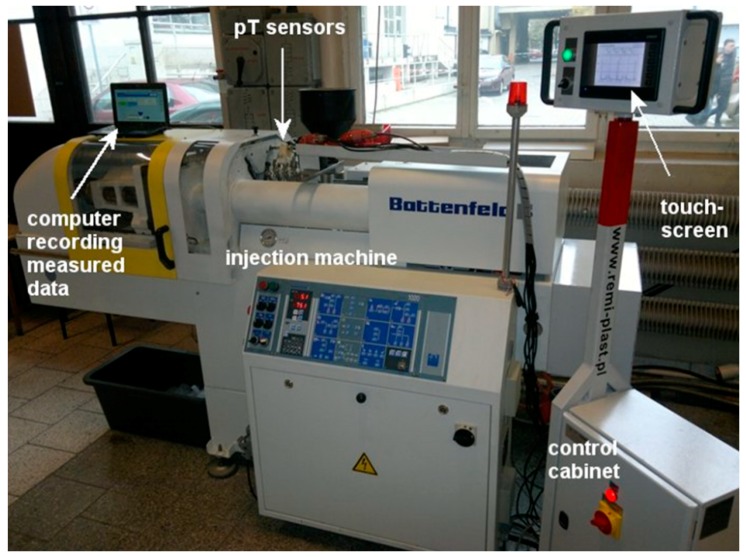
The test office for the plasticization process in the injection moulding.

The test office for the measurements of the output parameters of the plasticization process during the injection moulding consists of the suitably instrumented injection moulding machine linked to the collecting and processing data module and the computer for imaging and saving of the collected data. The test office shown in Figure 6 consists of:

1. the injection moulding machine, Battenfeld Plus 350/70,
2. four pressure/temperature sensors (analogue CDTAI200-1/2-1500-1-1-1J (Bagsik Sp. z o.o., Gliwice, Poland), range 0–150 MPa, 0–300 °C, OE: ±0.5% FS),
3. the torque—measuring device (analogue sensor DMF2X-250 (MEGATRON Elektronik GmbH & co. KG, Putzbrunn, Germany), range 0–250 Nm, OE: ±1% FS),
4. the inductive sensor for the screw rotational velocity measurements (induction detector E2A-S08KS02-WP-B1, Omron Corp, Kyoto, Japan),
5. the screw linear displacement sensor (analogue sensor LWH 0150 (Novotechnik U.S. Inc., Southborough, MA, USA), range 0–150 mm, LE: ±0.08%),
6. the control cabinet with the touch screen.

**Figure 7:**
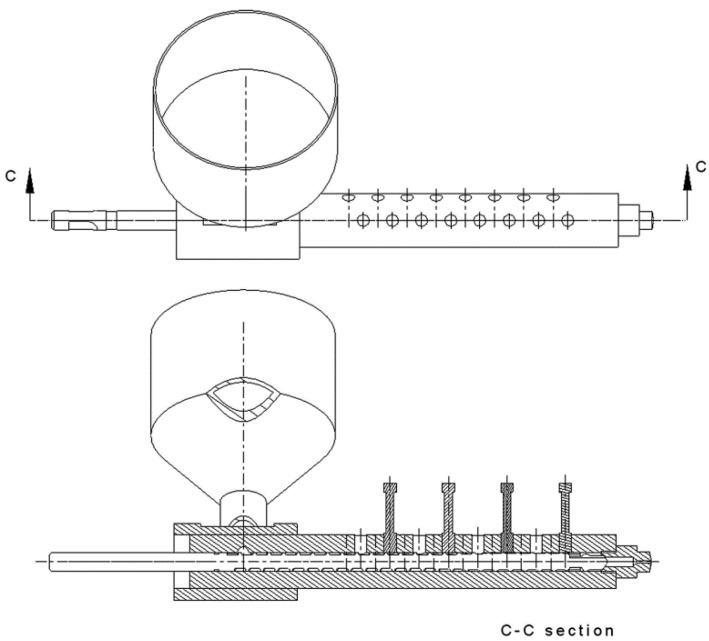
The arrangement of the holes in the barrel and the location of the pT sensors.

Table 1 shows the main features of the injection moulding machine used in the study. 16 holes for the pT sensors were made in the barrel. During the test, the pT sensors were placed in the holes no. 4, 8, 12 and 16 (see Figure 7). The hole no. 16 is located closest to the injection nozzle. Such a location of the sensors makes the pressure and temperature measurements on the maximal barrel length possible. In the maximal front position of the screw, the sensors are located over the 10, 14, 18 and 22-th screw coil, respectively (the injection screw has 22 coils). During the screw rotation phase, the screw moves back by the constant value of the screw stroke, which is equal to 2.5 coils.

The results presented below are related to the studies involving five typical thermoplastic polymers characterized in Table 2. Physical, thermal and rheological properties of the polymers necessary for the correct operation of the model were presented. The variable parameters of the injection moulding machine during the plasticization process study were the following:

1. the back pressure (changed in range of 4–24 MPa),
2. the screw velocity (changed in range of 30–70% of the maximal screw velocity),
3. the dwell time (changed in range of 8–50 s)—approximately equal to the cooling time of the product in the mould,
4. the average barrel temperature in the heated section.

Table 3 shows the values of the adjustable parameters of the injection moulding machine used in the experiments. Constant process parameters are shown in Table 4.

The studies on the plasticization process in injection moulding were carried out as two independent series of experiments:

1. The study by changing only one of the parameters listed in Table 3 and keeping constant the values of the other parameters, which were always equal to the third value in Table 3. The back pressure, the screw velocity and the dwell time were the same for all polymers. The symbols of T1–T5 were introduced due to the different average barrel temperature for processing of five polymers mentioned above. All three heating zones of the barrel were kept at the same (constant) temperature during experiments. This is because of the way the model works, where one average barrel temperature should be given. During the experiment, all the most important characteristics of the process were measured: the pressure and temperature profiles on the screw length, the torque on the screw, the power demanded to the heaters, the mass yield of the plasticization process and the recovery time.
2. The study by changing simultaneously two of the adjustable parameters listed in Table 3 and keeping constant the values of the other parameters, which were always equal to the third (middle) value from Table 3. During the test, the above-mentioned process characteristics were measured.

Because of the periodical (unsteady) character of the injection moulding process, all the results refer to the moment just before the end of the screw rotation. This moment corresponds to the maximum filling degree of screw channel with solid polymer and it is critical from the main plasticization characteristics point of view. It is of a fundamental importance for such quantities as the pressure and temperature profiles along the screw length, the power demand by the screw and the screw torque.

The pressure and temperature profiles for the variable input parameters: the back pressure, the screw rotational velocity, the dwell time and the barrel temperature are presented only for both extreme values of these parameters (for example, for the pT profiles determined with the variable parameter of back pressure as 3, 6, 10, 16 and 24 MPa, only the graphs for 3 and 24 MPa will be presented). The results for the intermediate values of the input parameters are not shown to keep the readability of the pictures, because these characteristics commonly change linearly with respect to the characteristics for extreme values.

Because there is a lot of results and they are very similar for different polymers in aspect of the shape of curves and the differences for the relevant theoretical and experimental characteristics, it was decided to present the output characteristics of one polymer for each variable process parameter. However, all studied polymers will be discussed for each analysed parameter. In order to standardize the charts, the characteristics obtained from the model are shown as thick lines without markers, while the experimental profiles represent the markers indicating the measurement points.

## 4. Results

The first part of the studies was related to change of one input process parameter, measurements of output characteristics and comparison with the characteristics determined by the model.

### 4.1. The Effect of Variable Back Pressure

**Figure 8:**
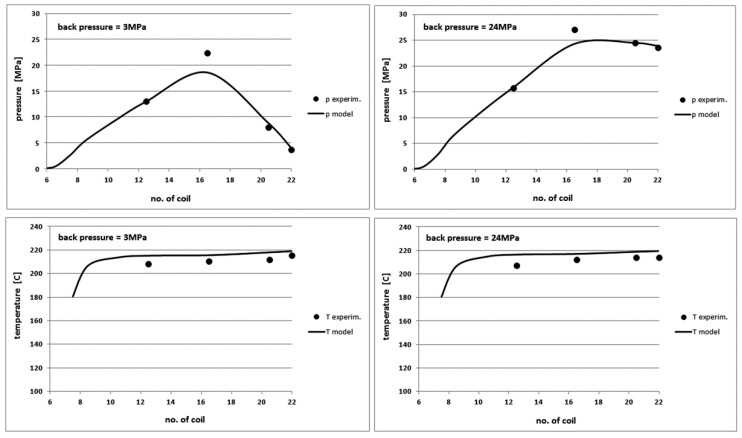
Comparison of theoretical and experimental characteristics of pressure (**top**) and temperature profiles (**bottom**) on the screw channel length in POM injection process for the back pressure of 3 MPa (**left**) and 24 MPa (**right**).

**Figure 9:**
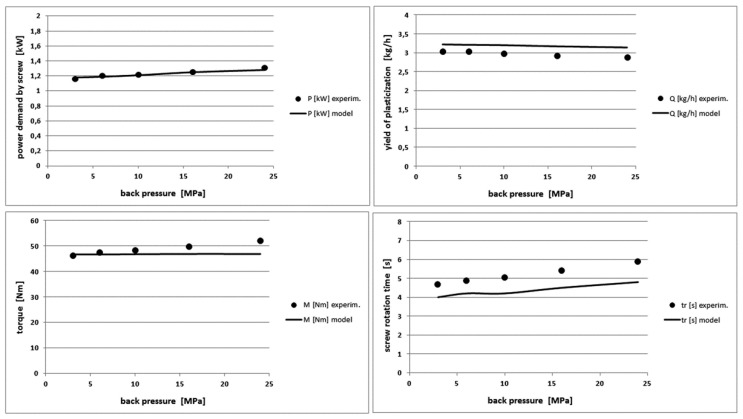
Comparison of other theoretical and experimental characteristics for different back pressure values in POM injection process.

Figure 8 shows the comparison of the pressure and temperature profiles along the screw length for POM. They were determined experimentally as well as were generated by the model for back pressure equal to 3 and 24 MPa, respectively. Figure 9 presents the other process characteristics such as the power demand by the screw, the mass yield of the plasticization process, the torque on the screw and the recovery time at changing back pressure.

The comparison of the theoretical and experimental data for the pressure and temperature profiles of the molten polymer shows a good agreement. The model slightly underestimates the pressure values and overestimates the temperature values by a few degrees for all polymers. These differences are the greater, the greater is the MFI of the polymer. We can also see a very small effect of the back pressure on the power demanded to the screw during its reciprocating motion, the process efficiency and the torque on the screw. The model predicts the values of these characteristics with a good accuracy. The model overestimates insignificantly the values of the power, the yield and the torque for the other polymers. The compliance with the model is also good and differences do not exceed a maximum of 20%.

### 4.2. The Effect of Variable Screw Rotational Velocity

**Figure 10:**
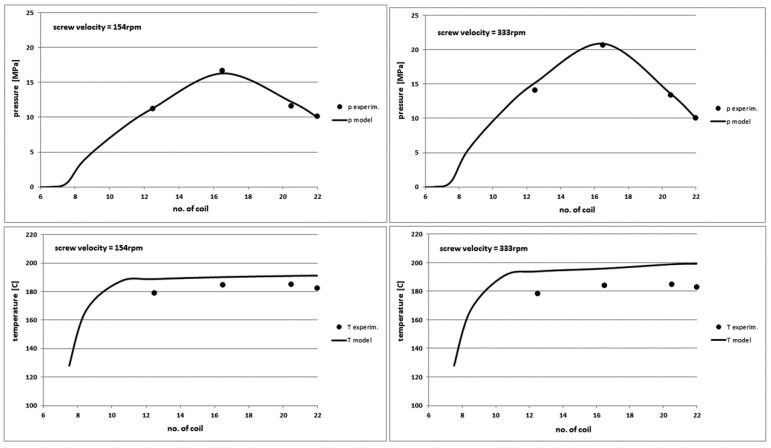
Comparison of theoretical and experimental characteristics of pressure (**top**) and temperature profiles (**bottom**) on the screw channel length in PE-LD injection process for the screw rotation velocity of 154 rpm (**left**) and 333 rpm (**right**).

The variable parameter of the rotational velocity of the screw brings the greatest dynamics of changes in output characteristics of the plasticization process of polymers during the injection moulding. An expected increase in the maximum pressure of the polymer with increasing screw rotational velocity is visible in Figure 10. The model predicts the pressure profile very well and it underestimates the pressure for other polymers by about 10–20%.

Differences in the temperature determining by the model are the greater, the greater is the rotational velocity of the screw. The differences do not exceed a few degrees for low screw velocity and they approach up to 20 °C for high screw velocity. This is probably caused by the overestimation of the effect of the viscous friction on the temperature increase of the polymer melt and it is related to the applied rheological model. However, an inaccurate measurement of the polymer temperature in the barrel can also be important. These issues will be discussed later.

**Figure 11:**
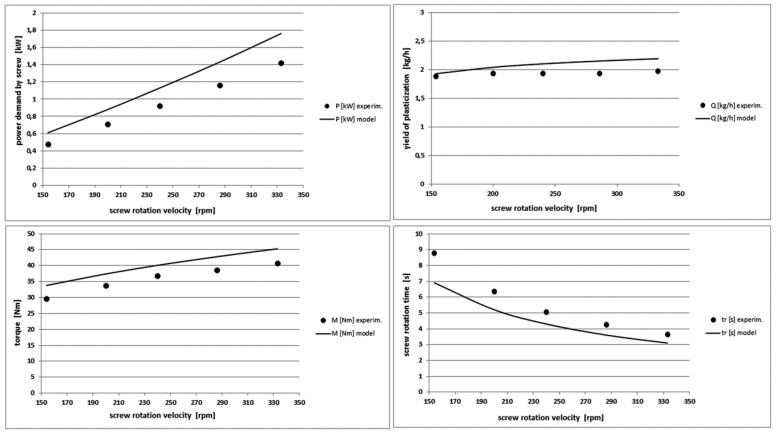
Comparison of other theoretical and experimental characteristics for different screw rotational velocity values in PE-LD injection process.

Figure 11 shows a sharp increase in the power demanded to the screw and a significant increase in the torque when the rotational velocity of the screw increases. The yield of the plasticization is practically constant, because the variable velocity of the screw rotation and therefore the variable time of its rotation does not affect practically the length of the injection cycle. Generally, the model predicts well the dynamics of changes of output characteristics and it overestimates them slightly. The maximum differences are for the power demand and they do not exceed 25%.

### 4.3. The Effect of Variable Dwell Time

**Figure 12:**
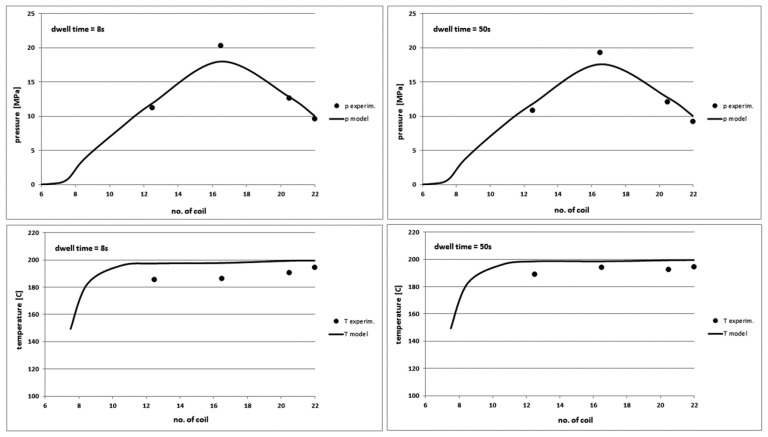
Comparison of theoretical and experimental characteristics of pressure (**top**) and temperature profiles (**bottom**) on the screw channel length in PE-HD injection process for the dwell time of 8 s (**left**) and 50 s (**right**).

**Figure 13:**
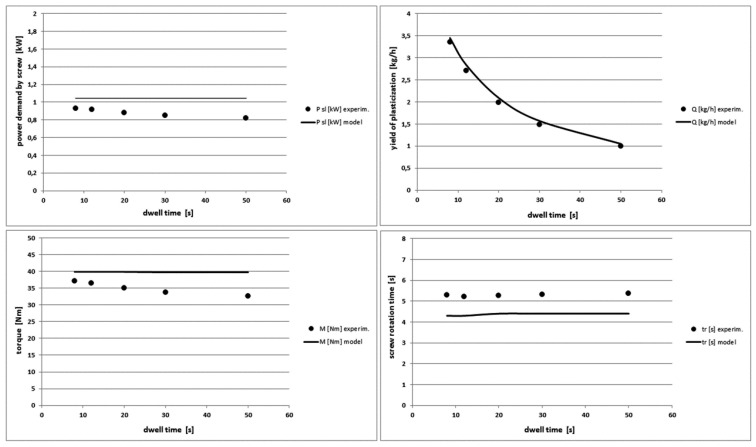
Comparison of other theoretical and experimental characteristics for different dwell time values in PE-HD injection process.

The dwell time parameter means exactly the downtime of the screw in the rear position and quantitatively is practically equal to the product cooling time in the injection mould. The dwell time does not significantly affect any output characteristics of the plasticization process except the process yield, as shown in Figure 12 and Figure 13. The presented model is completely insensitive to the change of this parameter. However, experimental studies indicate an increase of the polymer melt temperature and a very slight decrease in the power demand by the screw and the torque as the dwell time increases. This effect occurs because with the increase of the dwell time, the polymer stays longer in the heated barrel and its temperature slightly increases. This results in the lower viscosity, that is, the less flow resistance and the less power demand and torque. Furthermore, the model predicts well the plasticization yield.

### 4.4. The Effect of Variable Barrel Temperature

**Figure 14:**
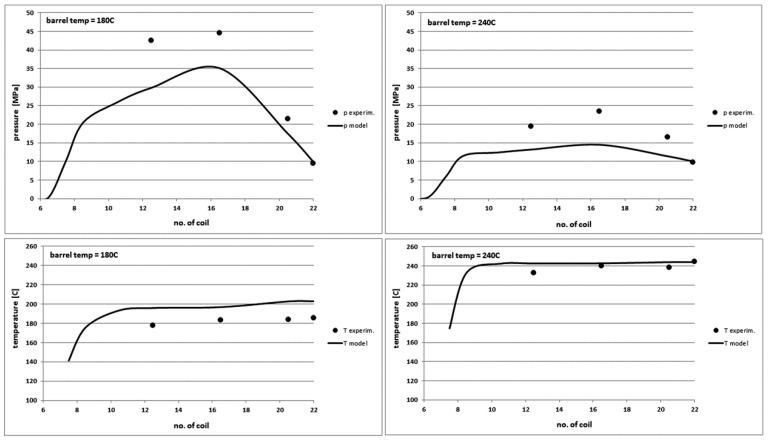
Comparison of theoretical and experimental characteristics of pressure (**top**) and temperature profiles (**bottom**) on the screw channel length in PS injection process for the mean barrel temperature of 180 °C (**left**) and 240 °C (**right**).

**Figure 15:**
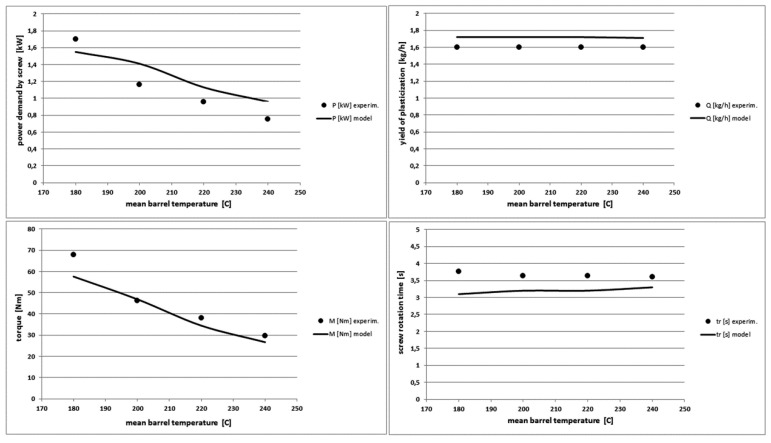
Comparison of other theoretical and experimental characteristics for different mean barrel temperature values in PS injection process.

We observe significant differences in the experimental and model pressure profiles in Figure 14. The model determines the pressure profiles at very low and high processing temperatures with less accuracy. For the other tested polymers, the pressure profile is overestimated at lower temperatures and at higher temperatures the polymer pressure is undervalued. The differences in extreme cases reach 40%. The model describes well the pressure profiles at average, typical processing temperatures. A more accurate analysis of the model showed that it is too sensitive for changes in the viscosity of molten polymers in the pressure calculation part. However, the model correctly determines the other characteristics of the plasticization process (Figure 15) with maximum differences not exceeding 20%.

As the temperature of the barrel increases, the power supplied to the screw decreases. However, the total power demanded to the plasticizing system of the injection moulding machine increases, which is related to the increase in the power demand by the barrel heaters [51].

The second part of the studies was related to the change of two input process parameters. At the same time, the rotational velocity of the screw and the back pressure in the tests were changed. These parameters are presented in Table 5. Other working parameters of the injection machine were constant, as in the first part of the studies. The tests were performed for PE-LD, PP and PS. The exemplary results are shown for PP.

**Figure 16:**
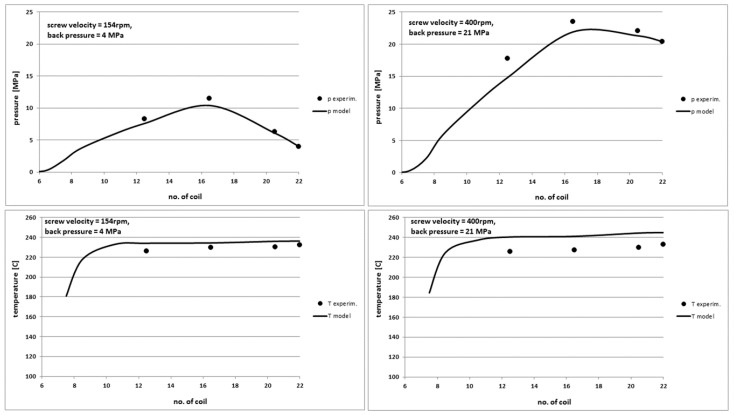
Comparison of theoretical and experimental characteristics of pressure (**top**) and temperature profiles (**bottom**) on the screw channel length in PP injection process for the screw rotational velocity of 154 rpm and the back pressure of 4 MPa (**left**) and 400 rpm and 21 MPa (**right**).

**Figure 17:**
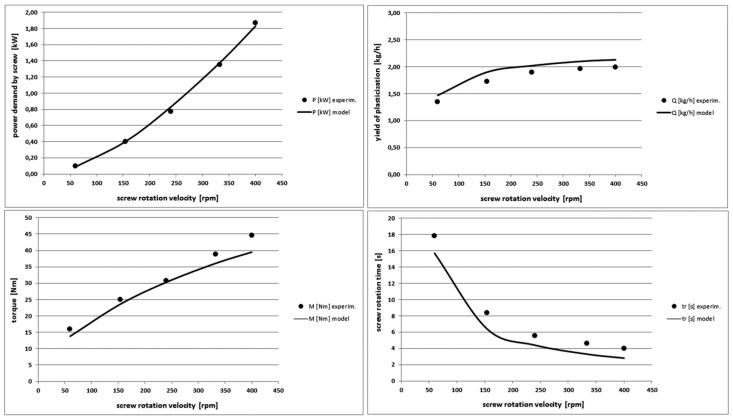
Comparison of other theoretical and experimental characteristics for different screw rotation velocity and back pressure values in PP injection process.

In Figure 16 and Figure 17 a comparison of the output characteristics for the injection moulding of PP is presented. We can see a very good agreement between the model curves and the experimental points. The model provides a very good pressure profile as well as the power demand, the torque and the process yield. Only the temperature profile is predicted by the model with the differences of approx. 5–7% for high screw rotational velocity. The same situation occurs for PE-LD and PS. In order to generalize these results, similar tests should be performed by changing other pairs of the injection working parameters. Research works in this area are continued.

## 5. Discussion

The mathematical model of the plasticization process in the injection moulding predicts values of the following characteristics:

(a) the pressure profile of the polymer in the screw channel,
(b) the temperature profile of the polymer in the screw channel,
(c) the profile of the solid polymer bed width in the screw channel (not verified),
(d) the power demanded by the screw in the plasticizing system,
(e) the torque on the screw,
(f) the yield of the plasticization process,
(g) the recovery time (or the injection cycle time),
(h) the SEC (the specific energy consumption).

The pressure of molten polymers in the screw channel is determined substantially correctly. The results of the comparison of the theoretical and experimental characteristics for all tested polymers indicate that the mean differences do not exceed 20%. Mostly, the model profiles cover with the experimental points very well. It is worth noting that the largest deviations from the real values are observed for very low rotational velocity of the screw and very low and high barrel temperature, that is, for the process conditions, which are very rarely used in practice. The model better predicts the behaviour of molten polymers in the conditions similar to those most commonly used in injection processing, that is, quite high rotational velocity and average, typical values of processing temperatures.

In order to better predict polymer pressure values, several problems will be analysed. The first problem to study in the near future is using a different rheological model. Two rheological models will be analysed:

1. the Carreau-Yasuda model, slightly modified by the authors [52],
2. the author’s rheological model based on the free volume theory:
  (31) $$\ln\frac{\eta }{\eta_{0}}=-\delta \;\tau^{m}$$
  where *δ*, *m*—the parameters of the equation, *η*_0_—the lower Newtonian viscosity.

The second factor, which will be analysed for improving the compliance of the pressure characteristics is the analysis of methods of polymer-metal friction coefficients determining. The values of the dynamic friction coefficients of the polymers used in this study were taken as the mean values from the literature and they were equal 0.1–0.2 for PP and PE-HD to 0.4–0.5 for PS and PE-LD. The pressure value of the solid polymer in the screw channel (which determines the pressure of the polymer melt) in the model significantly depends on friction coefficients values. It is known from the literature that the values of friction coefficients are variable and depends not only on the temperature but also on the pressure and the rotational velocity of the screw. In order to analyse this problem more closely, the apparatus for dynamic friction coefficients measuring for different polymers in variable temperatures, pressures (load) and a variable velocity of the movable wall will be constructed in the near future. This will allow to adapt the corrected values of the friction coefficients to the model. It is supposed to improve the compatibility of the model results with the experience.

Another problematic issue that appeared during the experimental research is the model of barrel feeding with the solid polymer during the injection process. In the previous studies authors assumed that in accordance with the known Tadmor model of the plasticization process in extrusion [3], a polymer granulate forms very quickly a solid block of material that moved forward due to frictional forces. Closer analysis of this problem and photos of the injection screw channel showed that loosely packed polymer granules are present in this form until they melt (more precisely: until the supersaturation of the polymer granulate with the melt will occur) [42]. On the other hand, when observing the work of the injection moulding machine it can be seen that during the rotation and the backward movement of the screw, the polymer granulate falling from the hopper fills the almost empty channel of the screw. Based on these simple observations it seems that the commonly used method of determining the pressure in the solid bed in the injection screw channel is not correct. It seems that a more adequate description of the injection barrel feeding with polymer granules is the starving feed, described quite recently by Wilczynski [21,22,23]. However, the work on this issue has not been developed yet for the injection moulding process. A simple approximation of this theory, implying the lack of pressure increase in the feed zone (pressure at the end of the zone was assumed to be equal to the pressure at the bottom of the hopper) resulted in a very low melt pressure in the barrel, which was too small in comparison to the experimental results.

The comparison of the temperature characteristics of molten polymers generally indicates that the model overestimates the real temperature values by approx. 2–15 °C. The greater are the differences between the theoretical and experimental profiles, the more viscous is the polymer. These differences increase as the rotational velocity of the screw increases. They decrease with the increase of the screw downtime as well as the barrel temperature increase and they are practically independent of the variable back pressure.

The temperature profiles determined experimentally are practically unchangeable for the different back pressures. The analysis of the model indicates that it overestimates the amount of the heat generated in the material due to the viscous friction. Moreover, large increases in the polymer temperature above the barrel temperature are not observed in the real plasticizing system of the injection moulding machine. Regardless of the polymer type and the process parameters, this increase always equals about 2–6 °C. This is thought-provoking because it seems that the extreme values of some operating parameters (a very high rotational velocity of the screw or a very high back pressure) should affect the temperature of the molten polymer in a way. Meanwhile, for a given polymer the temperature of the melt is practically constant in the same points of the barrel, regardless of the value of the screw velocity or the back pressure. Due to the above-mentioned facts, doubts relating to the method for measuring the temperature of the molten polymer in the screw channel appeared.

The polymer temperature measured by the pT sensor refers to the temperature of the polymer melt layer at the inner barrel wall. The model determines average temperature of the polymer melt in the entire cross section of the screw channel. This can be a source of differences, especially for a low fluidity of the material, when the viscous friction increases. It was decided to measure the temperature of molten polymers inside the screw channel in the future. We plan to place the tip of a thin thermocouple in the molten material in the screw channel through the hole in the barrel. The barrel already has 16 holes made for the pT sensors. After proper processing of locking bolts placed in the barrel holes, the thermocouple will be placed in the molten polymer on the depth of several mm into the screw channel, just after the end of the screw rotation. We also plan to carry out studies on a temperature of the solid polymer in the initial part of the barrel in the same way. It will verify whether temperature of the polymer melt inside the screw channel is comparable or different from the temperature of the polymer boundary layer. It will also enable to develop a mathematical model for determining the polymer temperature in the solid bed.

The methodology of the power demand and the torque determining has been corrected in the model in a relation to the works presented earlier [1,44]. As a result, the power and torque characteristics do not differ from the experimental ones by more than max. 20%. Moreover, an empirical correction was proposed to achieve better theoretical and experimental agreement, which, however, was not permanently adapted to the modified mathematical model. It is related to the limitation of the leakage flow for polymers with a degree of crystallinity c < 1. The mathematical model predicts well the power demand and torque values for the variable rotational velocity of the screw. It is practically insensitive to the changes in the dwell time and the back pressure, whereas the power demand and the torque increase slightly with the increase of the dwell time and the back pressure in the reality. In the case of the variable back pressure, the model and the experimental curves intersect somewhere about in the range of average values of the back pressure, that is, for most typical values of the plasticization process in the injection moulding. The corrected model also predicts well the power demand and the torque values for different barrel temperatures.

The yield of the process is very well determined by the model and the differences in theoretical and experimental values do not exceed a maximum of 10%. The model overestimates this parameter slightly in each case. This characteristic is closely connected with the next parameter determined by the mathematical model that is, with recovery time. This parameter is underestimated by the model by approx. 20%, that is, by about 1 s. It is caused by the method used to determine the recovery time by the measuring system. However, in practice, the value of the full injection cycle time is more important than the recovery time. The model also determines the cycle time. Thus, a typical recovery time error of 1 s/5 s = 20% means that the average error of the cycle time equals 1 s/20–30 s = 3–5%. It is therefore relatively small.

The model verification procedure should provide sensitivity analysis in addition to compliance tests. The sensitivity analysis involves examining changes in the output (variable) values of the model when the input parameters of the model change. It is required from a good model that small changes in the input parameters cause small changes in its output values only.

When an explicit algebraic equation describes the relationship between the independent variables and the dependent variable, the sensitivity analysis is easy to perform. In this case, the sensitivity coefficient SC, for a particular independent variable can be calculated from the partial derivative of the dependent variable with respect to the independent variable [53]:

(32) $$SC_{i}^{y}=\frac{x_{i}}{y}\;\frac{\partial y}{\partial x_{i}}$$

In simplification, it can be assumed that the sensitivity function (32) indicates how many percent will change the function value if the specified variable increases by 1%.

The matrix of results in the form of a table is presented in Table 6. It presents the results of changes in the size of the model variables (output data) with the increase of individual model parameters (input data) by 1%. An exemplary sensitivity test was performed for POM.

The model is sensitive to four parameters:

1. the temperature of the barrel,
2. the temperature of polymer melting,
3. the polymer-barrel friction coefficient,
4. the power-law exponent.

We can see the high sensitivity of the model to one of the parameters of the rheological equation (the power-law exponent *n*). On the one hand, this fact requires an accurate determination of the power-law equation parameters for each polymer used for the modelling process. On the other hand, it indicates the potential benefit of using another equation that describes the viscosity of molten polymers. However, as previously described, the applied power-law equation describes the molten polymer behaviour very well. It is indicated by the correct results of the model work. In addition, there is no guarantee that the model will be less sensitive to the parameters of another rheological model.

The second important parameter is the polymer-barrel friction coefficient. As mentioned earlier, the model is sensitive to the value of this parameter. For the needs of the model, coefficients of friction were determined as mean literature values from various sources. Perhaps the introduction of dependence of friction coefficients on temperature would still improve the model’s compliance with the experience.

## 6. Conclusions

The paper presents the comprehensive model of the plasticization process during the injection moulding. The experimental verification of the model using the specially designed and built research office was further described. The plasticization studies of five typical thermoplastic polymers during the injection moulding with different values of the back pressure, the screw rotational velocity, the dwell time and the barrel temperature were carried out. The values of experimental characteristics with the results generated by the simulation model were compared. It was found that the model correctly determines the dynamics of the plasticization process under the changes of the most important input parameters. The model predicts well the power demand and the torque values as well as the process yield. The average differences in the theoretical and experimental values do not exceed 10%. Slightly larger average differences (about 20%) occur in the determination of the pressure and temperature characteristics. It is worth noting, however, that the knowledge of the pT profiles is scientific and cognitive in nature. On the other hand, the results such as the power demand, the torque, the process yield and the SEC are of a practical importance. Here the model shows the results more compatible with the experience.

The model uses three groups of input parameters: geometrical parameters of the plasticizing system (mainly the screw), working parameters of the injection moulding machine and material parameters of the polymer being processed. In the experimental verification of the model, the second and third parameter groups are changed. All tests were conducted using one injection moulding machine and one screw. It is worth performing similar studies for a larger injection moulding machine as well as at least two different screws with the different length of feed, compression and metering zones to generalize or detail the results presented in this work.

## Figures and Tables

**Table 1:** Characteristics of the screw and the injection moulding machine.

screw diameter (mm)	25
relative screw length L/D	17
length of feed/melting/metering zone (coils)	14/4/4
channel depth in feed/metering zone (mm)	4.1/1.9
screw pitch (mm)	19
flight width (mm)	3.7
max. clamping force (kN)	350
max. injection volume, PS (cm^3^)	49
max. injection pressure (MPa)	157.5

**Table 2:** Material data for tested polymers.

Property	PE-LD	PE-HD	PP	POM	PS
type	Malen E FABS 23D022	Hostalen GC 7260	HP 515M	Schulaform 9A	Krasten 154
melt flow rate (g/10 min) ^b^	2.2	8.0	9.5	9.8	9.5
bulk density (kg/m^3^) ^a^	598	596	576	856	664
coefficient of dry friction (polymer-barrel) ^c^	0.5	0.4	0.3	0.2	0.3
coefficient of dry friction (polymer-screw) ^c^	0.4	0.3	0.2	0.1	0.2
density of solid (kg/m^3^) ^b^	920	960	907	1410	1050
specific heat of solid (J/(kg * K)) ^a^	2.35	1.82	1.75	1.71	1.24
thermal conductivity of solid (J/(m * s * K)) ^c^	0.32	0.43	0.21	0.29	0.16
melting temperature (°C) ^a^	114	137	168	170	93 *
melting enthalpy (kJ/kg) ^a^	113	202	93	155	-
average density of melt (kg/m^3^) ^a^	757	755	733	1150	963
average specific heat of melt (J/(kg * K)) ^a^	2.12	2.45	2.3	2.21	1.54
thermal conductivity of melt (J/(m * s * K)) ^c^	0.23	0.42	0.16	0.225	0.155
consistency coefficient (Pa s^n^) * 10^3 a^	51.9	7.9	15.7	4.8	230
power-law exponent ^a^	0.503	0.661	0.599	0.776	0.667
temperature coefficient (K^−1^) ^a^	0.0202	0.0114	0.0347	0.0176	0.0358

^a^ self-experimentally determined; ^b^ data from TDS; ^c^ averaged data from various literature sources; * flow temperature.

**Table 3:** Adjustable parameters of the injection moulding process.

	**Back Pressure (MPa)**				
	3	6	10	16	24
	**Screw Rotational Velocity (rpm)**				
	154	200	240	286	333
	**Dwell Time (s)**				
	8	12	20	30	50
	**Average Barrel Temperature (°C)**				
	T1	T2	T3	T4	T5
PE-LD	140	160	180	200	220
PE-HD	150	170	190	210	230
PP	190	210	230	250	270
POM	-	-	210	-	-
PS	-	180	200	220	240

**Table 4:** Constant parameters of the injection moulding process.

Parameter/Polymer	PE-LD	PE-HD	PP	POM	PS
injection pressure (MPa)	86	80	86	70	86
hold pressure (MPa)	47	42	47	35	28
hold time (s)	4	4	4	4	2.5
mould temperature (°C)	35	35	40	75	40

**Table 5:** Changed parameters in the second part of the studies.

Screw Rotation Velocity (rpm)	Back Pressure (MPa)
60	1.0
154	4
240	10
333	16
400	21

**Table 6:** The sensitivity test for POM.

Variables of the Model:	Power Demand (Screw)	Torque (Screw)	Yield	Cycle Time	Max Temp. of Melt	Max Pressure of Melt
**Input data (parameters):**						
Barrel velocity (*V_b_*)	1.5	0.6	0.1	−0.1	0.0	0.5
Temperature of barrel (*T_b_*)	−2.3	−2.7	0.0	0.0	0.8	−2.4
Dwell time (*t_d_*)	0.0	0.0	−0.7	0.7	0.0	0.0
Back pressure (*p_b_*)	0.0	0.0	0.0	0.0	0.0	0.2
Temperature of polymer melting (*T_m_*)	1.9	2.3	0.0	0.0	0.1	2.0
Polymer-barrel friction coefficient (*f_c_*)	1.7	1.7	0.1	−0.1	0.0	1.7
Bulk density (*ρ*_0_)	0.1	0.1	0.0	0.0	0.0	0.1
Density of melt (*ρ_m_*)	0.2	0.2	1.0	0.0	0.0	−0.3
Density of solid (*ρ_s_*)	0.0	0.0	0.0	0.0	0.0	0.4
Thermal conductivity of the molten polymer (*k_m_*)	0.0	0.0	0.0	0.0	0.0	0.0
Thermal conductivity of the solid polymer (*k_s_*)	0.0	0.0	0.0	0.0	0.0	0.0
Heat of fusion (*l*)	0.0	0.0	0.0	0.0	0.0	0.0
Specific heat of the molten polymer (*c_m_*)	0.2	0.2	0.0	0.0	0.0	0.1
Specific heat of the solid polymer (*c_s_*)	0.0	0.0	0.0	0.0	0.0	0.0
Consistency coefficient (*k*_0_)	0.7	0.7	0.0	0.0	0.0	0.7
Power-law exponent (*n*)	3.4	3.6	0.0	0.0	0.1	3.0
Temperature coefficient (*a*)	−0.6	−0.7	0.0	0.0	0.0	−0.5

## Explanations

back pressure	pressure in front of the screw during its rotation,
dwell time	time of polymer holding under pressure after mould filling,
recovery time	time of screw rotation.
